# CHANGES IN ADULT SIBLING CONFLICT AND OLDER PARENTS’ COGNITIVE IMPAIRMENT: THE MODERATING ROLE OF CARE PROVISION

**DOI:** 10.1093/geroni/igad104.0055

**Published:** 2023-12-21

**Authors:** Megan Gilligan, J Jill Suitor, Destiny Ogle

**Affiliations:** University of Missouri, Columbia, Missouri, United States; Purdue University, West Lafayette, Indiana, United States; Purdue University, West Lafayette, Indiana, United States

## Abstract

The life course concept of “linked lives” emphasizes that the life events of one family member have implications for other family members. Health events have been shown to be particularly salient in later-life families. We considered the consequences of older parents’ cognitive impairment on adult sibling relationship quality. Using mixed methods data from 327 adult children (nested within 96 families) collected as part of the Within-Family Differences Study (WFDS) T3, we examined the role of adult children’s care provision in the association between parents’ cognitive impairment and adult sibling tension. At T3 of the WFDS, the adult children were on average 60 years old and their parents were on average 90 years old. We conducted lagged multilevel linear regression modeling to account for nonindependence and to control for sibling tension seven years earlier before parents were experiencing cognitive impairment. The results of the quantitative analyses revealed that reports of sibling tension were higher in families in which parents experienced cognitive impairment. Moderation analyses indicated that the association between parents’ cognitive impairment and sibling tension was stronger for caregivers than noncaregivers. Qualitative analyses revealed that discrepancies in perceptions of the caregiving situation between caregivers and noncaregivers fueled sibling tension. Noncaregivers often seemed unaware of the caregiving situation or actively disengaged to avoid conflict. Taken together, these findings indicate that sibling tension is particularly high in the context of caring for older parents with cognitive impairment. In particular, adult children who are providing care are at increased risk of sibling tension.

